# Hyaluronic Acid in Female Reproductive Health: Tailoring Molecular Weight to Clinical Needs in Obstetric and Gynecological Fields

**DOI:** 10.3390/pharmaceutics17080991

**Published:** 2025-07-30

**Authors:** Giuseppina Porcaro, Ilenia Mappa, Francesco Leonforte, Giorgio Maria Baldini, Maria Francesca Guarneri, Marco La Verde, Felice Sorrentino, Antonio Simone Laganà

**Affiliations:** 1 Department of Gynecology and Obstetrics, Women’s Health Centre, 05100 Terni, Italy; 2 Department of Maternal and Child Urological Sciences, Sapienza University, 00161 Rome, Italy; 3 Public Health Physician, University Hospital Polyclinic “G. Rodolico San Marco”, 95124 Catania, Italy; 4 Obstetrics and Gynecology Unit, Department of Biomedical Sciences and Human Oncology, University of Bari “Aldo Moro”, 70121 Bari, Italy; 5 Azienda Sanitaria Provinciale of Palermo, 90141 Palermo, Italy; 6 Department of Woman, Child and General and Specialized Surgery, University of Campania “Luigi Vanvitelli”, 80138 Naples, Italy; marco.laverde@unicampania.it; 7 Department of Medical and Surgical Sciences, Institute of Obstetrics and Gynecology, University of Foggia, 71121 Foggia, Italy; felice.sorrentino.1983@gmail.com; 8 Unit of Obstetrics and Gynecology, “Paolo Giaccone” Hospital, Department of Health Promotion, Mother and Child Care, Internal Medicine and Medical Specialties (PROMISE), University of Palermo, 90127 Palermo, Italy; antoniosimone.lagana@unipa.it

**Keywords:** hyaluronic acid, high molecular weight, low molecular weight, physiological pregnancy, genitourinary syndrome of menopause, human papillomavirus infection, vaginal dryness

## Abstract

Hyaluronic acid (HA) is a ubiquitous glycosaminoglycan with distinct biological functions, dependent on its molecular weight. High-molecular-weight HA (HMWHA) primarily exhibits structural and anti-inflammatory roles, whereas low-(LMWHA) and very low-molecular-weight HAs (vLMWHA) actively participate in tissue regeneration and angiogenesis. This review highlights the pivotal roles of HA across the female reproductive lifespan, emphasizing how molecular weight dictates its therapeutic potential. In gynecology, LMWHA effectively alleviates symptoms of genitourinary syndrome of menopause, restores vaginal architecture, and mitigates complications following pelvic radiotherapy, improving both tissue integrity and patient quality of life. vLMWHA shows promise in enhancing viral clearance and lesion regression in human papillomavirus (HPV) infections. In obstetrics, HMWHA plays crucial roles in implantation, immunotolerance, and embryogenesis and maintains cervical barrier integrity to prevent ascending infections and preterm birth. Moreover, emerging clinical evidence supports oral HMWHA supplementation for reducing pregnancy complications, such as threatened miscarriage, subchorionic hematomas, and preterm delivery. This review underscores the necessity of considering HA’s molecular weight to optimize interventions in gynecological and obstetric care, offering tailored strategies to support women’s health throughout their lives.

## 1. Introduction

Hyaluronic acid (HA) has been of interest in various scientific fields since Meyer and Palmer isolated it for the first time in 1934 from bovine vitreous humor [1]. HA (also known as hyaluronan or sodium hyaluronate) is a natural polymer, constituting a fundamental component of the extracellular matrix (ECM), virtually in all human and animal tissues [2,3]. HA resides in the skin, synovial joint fluid, vitreous humor of the eye, heart valves, skeletal tissues, and the umbilical cord, where it represents the major component of Wharton’s jelly, along with chondroitin sulfate [4,5]. Although at first, it was considered only an inert “space-filler”, HA is now recognized as a pleiotropic molecule involved in an array of physiological processes, including cell proliferation, adhesion, migration, ECM remodeling, inflammation, embryogenesis, the maintenance of organ structural integrity, and angiogenesis [6]. For these reasons it has garnered significant interest from researchers across various sectors (food, cosmetic, pharmaceutical, and medical), increasing the interest in its therapeutic potential [7], and it has been recognized and approved by the FDA for the treatment of various inflammatory conditions.

Given the properties of hyaluronan and its extensive application in medicine, there is significant interest in optimizing the HA production process to meet high quality standards. Historically, HA for biomedical use was extracted from animal sources, such as rooster combs or bovine vitreous humor. However, limitations in raw material availability have driven the shift toward alternative production strategies [8]. Commercial HA is primarily obtained through microbial fermentation, although this approach necessitates costly downstream purification due to the pathogenic nature of most native HA-producing organisms [9]. Metabolic engineering currently represents an interesting opportunity to obtain HA from nonpathogenic GRAS (generally regarded as safe) microorganisms. To overcome these limits, endotoxin-free HA has already been synthesized via recombinant hosts including *Lactococcus lactis* [10], *Bacillus subtilis* [11], *Escherichia coli* [12], and *Corynebacterium glutamicum* [13], and the introduction of bioreactors with modified bacteria allows for the production of highly pure HA without contaminants.

**Figure 1:**
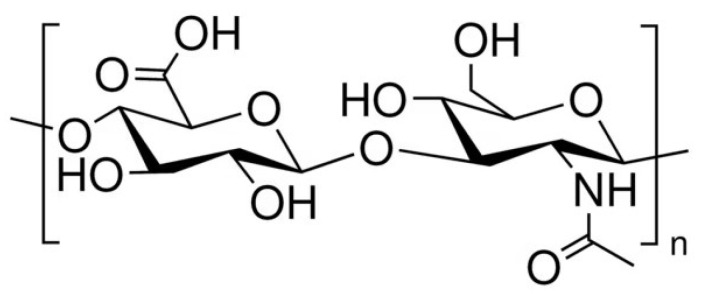
The chemical structure of hyaluronic acid.

HA is a non-sulfated glycosaminoglycan with a regular structure composed of repeated disaccharide units of D-glucuronic acid and N-acetyl-D-glucosamine, linked by β-1,4 and β-1,3 glycosidic bonds (Figure 1). Its turnover is finely regulated through a balance between biosynthesis and degradation. [14,15]. Three isoforms of membrane-bound glycosyltransferases—hyaluronan synthases (HAS1, HAS2, and HAS3)—catalyze the polymerization of HA chains on the inner surface of the plasma membrane. Conversely, hyaluronidases (HYALs) and oxidative mechanisms mediate its catabolism [15,16]. The molecular weight depends on the activity of the abovementioned hyaluronan synthase enzymes (responsible for the polymerization of HA resulting in HA chains of varying lengths), the extent of degradation via hyaluronidases (which fragment the polymer into smaller oligosaccharides), and the actions of reactive oxygen species (that can break glycosidic bonds and generate low-molecular-weight fragments). High- and low-molecular-weight forms exhibit distinct, sometimes opposing physiological activities [17,18].

High-molecular-weight hyaluronic acid (HMWHA; ≥500 kDa) is an essential structural framework for cellular functions, thus offering a secure matrix. It is a component of healthy adult tissues, like skin and joints. It has anti-angiogenic and anti-inflammatory activity, and it is a natural immunologic depressant. Moreover, HMWHA hygroscopic properties attract water molecules, creating a hydrated environment that facilitates nutrient exchange and enhances cell motility. Furthermore, it lubricates cells and makes them move more quickly. The large size and branching structure of HMWHA facilitates lubrication and hydration [19,20].

Low-molecular-weight (LMWHA; 100–400 kDa) or very low-molecular-weight (vLMWHA; <10 kDa) [21] are extremely useful in tissue regeneration and wound healing [22,23]. They possess immunostimulatory and angiogenic activity, which is necessary to form new blood vessels, provide oxygen and nutrients, and heal tissue [23]. Furthermore, specific fragments exhibit antibacterial properties, which may aid in infection control and contribute to an optimal wound-healing environment [24].

In recent years, several papers have addressed this issue—the distinction between high- and low-molecular-weight HA—within various medical fields, especially regarding joint problems (such as knee osteoarthritis) [25], dermatology [26], multifunctional therapeutic systems [27], and regenerative medicine [28]. Its roles in extracellular matrix homeostasis, hydration, and tissue remodeling underpin various physiological processes, with bioactivities that differentially modulate inflammatory, immunological, and cellular pathways, with actions depending on the molecular weight. A significant amount of growing evidence has also demonstrated that HA can exert pivotal functions within the female reproductive tract across the lifespan, encompassing youth, reproductive age, pregnancy, and senescence [29,30,31,32,33,34]. However, in obstetrics and gynecology, these molecular weight distinctions are often underappreciated, leading to conceptual ambiguities regarding its therapeutic applications.

Therefore, the purpose of this review is to more clearly define the molecular weight of HA (high, low, or very low) and the reasons that, based on various clinical data, it is more suitable for use in medical applications.

## 2. Supplementation of Low-Molecular-Weight (LMWHA) and Very Low-Molecular-Weight Hyaluronic Acid (vLMWHA) in Gynecological Disorders

### 2.1. LMWHA Is Useful in the Case of Genitourinary Syndrome of Menopause (GSM)

The healthy vaginal [35,36] mucosa is a multi-stratified epithelium covered by a “glycocalyx”, a glycan coat that lubricates the lower tract of the vagina during sexual intercourse, provides physical protection from pathogens, mediates host–microbe interactions, retains immunoglobulins and antimicrobial peptides, and harbors numerous microorganisms [37,38]. The vaginal microbiota is particularly abundant in *Lactobacillus* species, which protect the female genital tract from multiple conditions. *Lactobacillus* species are in fact essential in converting the cytoplasmic stock of glycogen into lactic acid in the stratum corneum, and in maintaining a hostile vaginal ecosystem (pH 3.5–4.5) for pathogenic bacterial and viral species [39,40]. Hormone changes throughout the life cycles of women influence the vaginal microbiome from birth to post menopause. During the reproductive years, the presence of a microbial community dominated by *Lactobacillus* species is associated with a lower pH and lower risk of bacterial vaginosis (BV) and sexually transmitted infections [41]. However, *Lactobacillus* species abundance declines during menopause and after it [42]. It is estimated, for example, that 27% to 84% of postmenopausal women suffer from genitourinary syndrome of menopause (GSM), a set of symptoms and signs resulting from the effects of estrogen deficiency. Principal symptoms include vaginal dryness, painful sex, burning, and dysuria. GSM is generally progressive without effective therapy, and it often negatively impacts on a woman’s sexual health and quality of life (QOL) [43]. Therefore, the preservation or restoration of the vaginal mucosa’s structure is extremely important for maintaining functionality, general well-being, and preventing sexually transmitted infections.

LMWHA (<500 kDa) and vLMWHA (<10 kDa) are useful for fulfilling these outcomes (Table 1). Essendoubi, M. et al. [44] tested HA with different molecular weights to demonstrate which molecular weight had a better absorption when locally applied. The authors showed that LMWHA (20–300 kDa) passed through the stratum corneum of the skin, in contrast to the impermeability of HMWHA (1000–1400 kDa) [44]. Moreover, Damodarasamy, M. et al. [45] determined that LMWHA (250 kDA) could significantly accelerate skin healing by increasing the expression of collagen synthesis compared to HMWHA, a fundamental component of the vaginal mucosa [45].

Costantino, D. et al. [46] assessed the efficacy and safety of LMWHA vaginal suppositories in a cohort of 150 postmenopausal women presenting with urogenital atrophy. Throughout the treatment period, no adverse effects were reported. The intervention yielded markedly positive outcomes in terms of symptom relief, with the local administration of LMWHA facilitating enhanced mucosal absorption and significantly ameliorating severe manifestations such as vaginal dryness, pruritus, burning sensations, and dyspareunia [46]. LMWHA is also directly effective in restoring the physiological architecture of a compromised vaginal mucosa [47]. La Galia et al. [47] investigated the effect of the oral administration of LMWHA in patients with atrophic vaginitis. The study enrolled a total of 12 women, aged 45–65 years, who were at least 12 months postmenopausal and had symptoms of atrophic vaginitis, and they received vLMWHA for three months. The results showed that vLMWHA significantly (*p* < 0.001) increased the number of epithelial cell layers and the overall thickness of the vaginal epithelium compared to baseline measurements. Notably, the histological analysis revealed a substantial augmentation in epithelial stratification post intervention [47]. Furthermore, the combined use of oral supplementation and vaginal suppositories may constitute a valid synergistic strategy to achieve sustained symptomatic improvement, thus enhancing patients’ quality of life [48].

Until the 1990s, hormone replacement therapy (HRT), either systemic or topical, was often used for the treatment of menopausal effects on the urogenital tract, including those related to vaginal atrophy [49].

It has been used in clinical practice for over 60 years since the 1960s; however, the benefits and risks of HRT have been controversial. The study published in 2005, carried out on approximately one million postmenopausal women treated with HRT, reported a significant increase in the incidence of endometrial cancer in women treated with estrogens alone or a combined therapy (estrogens plus progestin compounds) [50]. Upon further analysis of the WHI data and with support from newer studies, international societies have formulated guidelines and announced consensus opinions on the use of HRT [51]. Low-dose vaginal estrogen therapies include various preparations, and studies have demonstrated that those therapies are an effective and safe treatment for GSM. Nevertheless, given that low-dose vaginal estrogens may restrict but not eliminate the absorption of estrogen, an alternative approach may be useful for those women with breast or endometrial cancer. Moreover, many women still prioritize complementary and alternative therapies, especially those worried about potential risks or with contraindications for HRT [51].

HA may represent a valid alternative to preparations containing estradiol, estradiol valerate, or conjugated estrogens. A multicenter, randomized, controlled, clinical trial evidenced the efficacy and safety of HA compared with estriol cream in postmenopausal women under the age of 70 with vaginal dryness [52]. HA significantly improved clinical symptoms after 30 days of treatment, without adverse effects [52]. Another five-year, double-blind, placebo-controlled clinical study compared the administration of the combination of hyaluronic acid and isoflavones in menopausal women for the treatment of the symptoms of menopause—urogenital atrophy and osteoporosis in relation to existing hormone replacement therapies. No differences were observed between the group taking HRT and the group treated with hyaluronic acid in terms of the improvement of symptoms in relation to the start of treatment, thus supporting that hyaluronic acid may have the same favorable effect as the administration of HRT [53].

Moreover, HA emerges as a valuable therapeutic option in women in breast cancer survivors [54]. In these women, HRT is contraindicated, and often, both clinicians and patients are reluctant to use topical estrogen treatments [55]. Thus, HA emerges as a valuable therapeutic option also in this context, leading to significant improvements in urogenital atrophy, quality of life, sexual health, and even urinary incontinence in post-breast cancer patients, outperforming hormone treatments in certain outcomes [56].

### 2.2. LMWHA Useful Against Pelvic Radiotherapy Discomfort

LMWHA has demonstrated the ability to accelerate tissue healing processes [57], which is particularly relevant in cases where the vaginal mucosa is compromised by invasive treatments, such as radiotherapy (RT). RT, frequently employed in pelvic, rectal, and cervical malignancies, inevitably induces diffuse inflammatory responses, leading to adverse effects including irritation, edema, burning, itching, vaginal atrophy, and dyspareunia, thereby diminishing patients’ quality of life and treatment compliance [58]. LMWHA represents an interesting approach to mitigate these complications. A prospective randomized study investigated the efficacy of LMWHA vaginal suppositories (MW 100–400 kDa) administered over four months concomitantly with pelvic RT and brachytherapy (BRT) in patients with cervical cancer (CC). Treatment with LMWHA induced a marked decrease in vaginal atrophy and inflammation, cell atypia, mucous edema, epithelial acanthosis, and fibrosis compared with the control group [59].

Vaginal stenosis (VS), characterized by a narrowing and/or shortening of the vaginal canal, is a common consequence of RT and contributes to sexual dysfunction and dyspareunia, with lasting repercussions on quality of life [60,61]. While its standard management involves vaginal dilators to prevent scar formation [62], this approach is limited by questionable efficacy and poor patient adherence due to its chronic nature. LMWHA vaginal suppositories (MW 100–400 kDa) alongside standard care may offer additional benefits, including improvement in emotional well-being [63]. Delia et al. [63] reported that, compared to controls who experienced moderate to severe symptoms at follow-up, nearly 90% of patients receiving LMWHA during RT for cervical cancer reported either an absence of or only mild symptoms, notably regarding dryness, inflammation, and dyspareunia [63].

### 2.3. vLMWHA Is Useful in Human Papilloma Virus (HPV) Infection

An interesting application of oral vLMWHA, is in human papillomavirus (HPV) infection. HPVs are the primary etiological agents for several cancers, including cervical cancer [64]. HPV infection is mainly acquired through sexual intercourse [65]. The virus spreads during sexual interaction through close skin-to-skin contact, which preferably involves areas that receive trauma and/or minor injuries. Indeed, the loss of continuity in the epithelia lets the virus particles get through and infect the cells. Therefore, restoring the integrity of cervical tissues may represent one of the goals for preventing HPV infections. vLMWHA may contribute to reaching this target. Frega, A. et al. [66] first demonstrated the additive effect of vLMWHA in stimulating apoptosis in a model of HPV-infected cell lines. The results evidenced that vLMWHA in association with epigallocatechin gallate (EGCG), folic acid (FA), and vitamin B12 (B12) induced a significant increase in apoptosis and p53 gene expression in HPV-infected cells and concomitantly decreased viral E6/E7 gene expression [66].

Moreover, five clinical studies investigated the antiviral effects of vLMWHA in combination with EGCG, FA, and B12. One case report showed complete HPV clearance in a young, fertile woman with a nine-year history of HPV persistence. The patient, despite many Loop Electrosurgical Excision Procedures, still suffered from HPV lesions. The study demonstrated that after only two months of treatment with two tablets per day of such combined molecules, preexisting HPV high-grade lesions (HSILs) and HPV low-grade lesions (LSILs) significantly improved. In addition, a 6-month period of follow-up confirmed the negativity to HPV infection and normal cytological result [67]. A subsequent publication reported the improvement in persistent HPV infection in five clinical cases, also including anal HPV infection. The oral treatment, with one or two tablets per day, for 6 or 3 months, respectively, according to the individual clinical history of the patient, revealed HPV clearance in all five patients, also resolving pre-existing cervical lesions [68].

A clinical study by Aragona, C. et al. [69] evaluated the effect of vLMWHA + EGCG + FA + B12 treatment on 20 women affected by LSILs and HPV persistence for two or more years compared to a control group with comparable clinical characteristics. The authors observed a viral clearance rate and lesion regression in 85% of treated patients compared to 25% in the control group (*p* = 0.000137) after 3 months [69]. Moreover, a clinical study conducted by Tinelli, A. et al. [70] on 163 women with HPV infection demonstrated that approximately 81% of patients treated with vLMWHA, in combination with EGCG + FA + B12, achieved HPV clearance after 3 months, compared to 60% in the untreated control group evaluated at the same timepoint. The treatment also improved LSILs in about 41% of the treated patients, compared to 9% of cases in the control group. In addition, after 6 months, approximately 84% of treated patients reported physiological cytological results, compared to about 37% in the control group [70].

Finally, a recent clinical study involving a larger number of patients (*n* = 106) confirmed that a 6-month treatment with vLMWHA in combination with EGCG + FA + B12 both cleared the viral infection and resolved baseline lesions in about 85.8% and 92.3% of patients, respectively. [71].

**Table 1:** Clinical evidence supporting the use of LMWHA in gynecological disorders.

Study	Study	Patients	Treatment	Outcomes	Ref.
Costantino, D. et al., 2008	Open-label, non-controlled clinical trial	Total *n* = 150 postmenopausal women	Vaginal suppositories (5 mg of LMWHA/suppository), one suppository for 28 days total	Decrease in vaginal dryness and itching, burning, and dyspareunia symptoms	[46]
La Galia et al., 2014	Randomized, placebo- controlled clinical trial	Total *n* = 12 women with atrophic vaginitis (*n* = 6 treatment group; *n* = 6 placebo group)	Oral tablets (200 mg of vLMWHA/tablet), two tablets/day for 10 days; subsequently one tablet/day for three months	Increase in epithelium thickness and number of epithelial layers Decrease in itching, burning, and dyspareunia symptoms	[47]
Prestia, V.M. et al., 2020	Non-controlled clinical trial	Total *n* = 50 menopausal women	One oral tablet (100 mg of LMWHA) and one suppository per day (5 mg of LMWHA) for 5 weeks; then one tablet per day for 10 months	Decrease in vaginal dryness and burning, itching, and dyspareunia perceptions	[48]
Chen, J. et al., 2013	Multicenter, randomized, controlled, open-label, parallel-group clinical trial	Total *n*= 144 postmenopausal women (*n* = 72 treatment group; *n* = 72 control group)	Vaginal gel of LMWHA once every 3 days for a total of 10 applications over 30 days	Increase in vaginal dryness and itching, dyspareunia, and burning sensations	[52]
Jokar, A. et al., 2016	Randomized, controlled clinical trial	Total *n* = 56 menopausal women (*n* = 28 treatment group; *n* = 28 = control group)	Vaginal cream (containing 5 mg of hyaluronic acid) for 8 weeks	Decrease in urinary incontinence, vaginal dryness, itching, and dyspareunia	[56]
Dinicola, S. et al., 2015	Prospective, randomized clinical trial	Total *n* = 45 women in treatment with RT and BRT for cervical cancer (*n* = 23 treatment group; *n* = 22 control group)	Two vaginal suppositories (5 mg of LMWHA/suppository) for 4 months	Improvement in inflammation, cell atypia, fibrosis, mucositis, and bleeding	[59]
Delia, P. et al., 2019	Randomized clinical trial	Total *n* = 180 women undergoing radiotherapy for CC (*n* = 88 treatment group; *n* = 89 control group)	Vaginal suppositories (5 mg of LMWHA/suppository) for 5 weeks	Lower intensity of pain; decrease in vaginal dryness, inflammation, and dyspareunia	[63]
Grandi, G. et al., 2022	Case report	One patient with persistent HPV infection	Oral tabs (containing 50 mg of vLMWHA)	Increase in HPV lesions and viral clearance	[67]
Calcagno et al., 2024	Case report	Total *n* = 5 patients with persistent HPV infection	Oral tabs (containing 50 mg of vLMWHA)	Increase in HPV lesions and viral clearance	[68]
Aragona et al., 2023	Open-label, controlled clinical trial	Total *n* = 41 women with persistent HPV infection (*n* = 20 treatment group; *n* = 21 control group)	Oral tabs (containing 50 mg of vLMWHA)	Increase in HPV lesions and viral clearance	[69]
Tinelli, A. et al., 2024	Open-label, controlled clinical trial	Total *n* = 163 women with HPV infection (*n* = 86 treatment group; *n* = 77 control group)	Oral tabs (containing 50 mg of vLMWHA)	Increase in HPV lesions and viral clearance	[70]
Porcaro, G. et al., 2025	Non-controlled clinical trial	Total *n* = 106 women with HPV infection	Oral tabs (containing 50 mg of vLMWHA)	Increase in HPV lesions and viral clearance	[71]

RT = radiotherapy; BRT = brachytherapy; and CC = cervical cancer.

## 3. Role of High-Molecular-Weight Hyaluronic Acid (HMWHA) in Obstetric Field

### 3.1. HMWHA Supports Physiological Pregnancy

HA involvement in pregnancy and throughout the gestational period has been an area under increasing investigation for over 30 years. Being present in all major tissues of pregnancy (uterus, cervix, placenta, decidua, chorion, amnion, and ovary), HMWHA is involved in all the required processes to maintain a physiological pregnancy [17,72,73,74] (Table 2).

HMWHA is of paramount importance during pregnancy, from conception to full of gestation, since it is either a structural or a regulatory molecule. HA is a prominent component of ECM, involved in tissue growing and remodeling. The HA-enriched transfer medium improved pregnancy and implantation rates in patients with multiple embryo transfer failures, suggesting that HMWHA is essential for embryo implantation and pregnancy evolution [75]. In early pregnancy, high concentrations of HMWHA, secreted by Decidual Stromal Cells (DSCs) and human trophoblasts, are essential for embryo implantation and pregnancy establishment [76]. The interaction between HMWHA and its receptor CD44 induces the proliferation of DSCs, inhibits their apoptosis, and improves proliferation and trophoblast migration [76].

Pregnancy is a unique immunological challenge; therefore, immunotolerance is essential for a successful outcome. Although all variants of HA are important for their specific activities, HMWHA, with its immunomodulatory properties, is fundamental in modulating the immune response at the maternal/fetal interface [73]. HMWHA induces the secretion of anti-inflammatory cytokines such as IL-10 [74]; inhibits the expression of pro-inflammatory factors, such as TNF-α or IFN-γ [77], which can induce pregnancy complications (recurrent miscarriage, premature rupture of membrane, and preeclampsia) [78]; and supports maternal immunotolerance by stimulating the cell differentiation of T-naive into Treg, and decidual macrophages into M2, at the maternal–fetal interface [79].

### 3.2. HMWHA Supports Embryonic Development

HMWHA is also crucial in embryogenesis, regulating extracellular matrix expansion, cell migration, and organ morphogenesis.

It is indispensable for cardiac cushion formation and endothelial-to-mesenchymal transition during heart development. During the early stages of heart development, HMWHA is a necessary component of the extracellular supramolecular array expanding the ECM, a requisite for the formation of cardiac jelly and endocardial cushions [80]. In E9.5 Has2^−/−^ (Has2 null) mouse embryos, cardiac cushions do not expand, and the embryos die between E9.5 and E10.0, since HMWHA synthesis is abrogated [80]. In the absence of HMWHA synthesis, the endothelial-to-mesenchymal transition during heart development does not occur [80]. Moreover, embryos lacking HMWHA synthesis have a low number of red blood cells, a lack of vitelline vessels in the yolk sac, and severe cardiac and vascular abnormalities [80].

Hyaluronan also has a pivotal role in intestinal morphogenesis [81]. Gut looping is a highly conserved process within species, and every individual should have the same looping patterns [82]. Deviations from the prescribed looping pattern (intestinal malrotation) predisposes individuals to volvulus, the self-strangulation of the gut [83]. Experiments in chickens and mice embryos evidenced that the rotation is driven by the dorsal mesentery (DM), a mesodermal structure that suspends the gut tube along the dorsal body wall [81]. In particular, the accumulation of HMWHA on the right side of the DM increases ~12 fold compared to on the left side [81,84]; moreover, its accumulation is also accompanied by covalent HC peptide modifications driven by the glycoprotein tumor necrosis factor-stimulated gene-6 (TSG-6), an important component of the ECM containing a hyaluronan-binding site. The accumulation of HA on the right side and the Tsg6-HA pathway on the right side, not on the left one, triggers the dramatic expansion of the right side of the DM, driving midgut rotation and vascular gut development. In Tsg6^−/−^ embryos, midgut malrotation and volvulus occur, thus evidencing once again that the right-side-restricted and ECM-derived pathway initiated by HMWHA is peculiar in intestinal morphogenesis [81].

Prior to right-sided expansion and gut tilting, there are two lines of endothelial cells in the DM, one on each side, with the capacity for differentiating into blood vessels. However, as the right side expands, the right-sided endothelial cells disperse, leaving only the left-sided endothelial cells to form a key gut artery [81]. This artery later branches and goes on to form the ileocolic and middle colic arteries, which supply a large portion of the adult intestine with blood. [81]. Asymmetry in the DM with HA-rich ECM as a key spatiotemporal modulator evidences how important the role of HMWHA is in employing distinct downstream signaling mechanisms to drive ECM expansion and vascular exclusion during gut looping [81].

Craniofacial morphogenesis depends on the correct migration of cranial neural crest cells (NCCs). The cranial neural crest is composed of a migratory population of multipotent cells arising at the edge of the neural tube. During neurulation, NCCs migrate into the branchial arches and contribute to various cell types, such as cranial ganglia, pigmented cells, craniofacial mesenchyme, and head skeletal elements.

Gene expression analysis has evidenced that the ECM surrounding the NCCs is rich in hyaluronan. The presence of HMWHA is necessary for both post-migration cranial NCC survival and their skeletogenic differentiation [85]. HMWHA synthetized via HAS2 is also indispensable for palatal morphogenesis [86]. HMWHA accumulation in the palatal shelves increases the ECM space to cause shelf expansion, which drives palatal shelf movement, but also instigates the interaction between the palatal shelves and tongue during shelf elevation. Has2 ko mutant mice showed severe orofacial anomalies including micrognathia, tongue protrusion, and complete cleft palate [86]. Hyaluronan also has a peculiar role in skeletal limb development [87]. Indeed, as evidenced in experiments using Has2-deficient mice, limbs are severely shortened and the joint regions are larger than normal and lack distinct joint cavities, thus indicating that HA plays a crucial role in skeletal growth, patterning, chondrocyte maturation, and synovial joint formation [87]. The next critical step in joint formation is a cavitation process, resulting in the formation of a cell-free fluid-filled space within the interzone, which represents the future synovial cavity that leads to the separation of adjacent skeletal elements. In Has2-deficient mice’s limbs, the cavitation process that gives rise to the synovial cavities is defective [88].

### 3.3. HMWHA Supports Endogenous Progesterone (P4)

HMWHA is essential in supporting the activity of endogenous progesterone (P4) [89]. P4 is a key regulator of membrane homeostasis during physiological gestation. The placenta sustains P4 production, and its functional decrease seems to induce parturition [90,91]. P4 inhibits myometrial contractility through genomic and non-genomic pathways: classic nuclear receptors (nPRs), functioning as ligand-activated transcription factors, mediate genomic pathways, while membrane-bound PRs mediate non-genomic pathways for a rapid response [92]. PGRMC1 [93,94] is involved in several biological functions including steroidogenesis and cellular homeostasis [95] and mediates the anti-apoptotic effects of P4 on granulosa cells (GCs), which provide physical support and the microenvironment required for oocyte development [96,97]. Several authors have also reported that PGRMC1 may mediate the inhibition action of P4 on the myometrium’s contractility [98]. PGRMC1 is in fact expressed in all layers of the fetal membrane (amnion, chorion, and decidua) and in the myometrium during pregnancy [99]. It is essential in supporting pregnancy, since its lower expression is associated with preterm labor (PTL) and the preterm premature rupture of membranes (PPROM) with or without histological chorioamnionitis (HCA) [100].

Scientific evidence has demonstrated that HMWHA increases the expression of PGRMC1 in a time- and concentration-dependent manner [89]. Zhao et al. showed that PGRMC1 expression was correlated with the level of HMWHA in primary ovarian insufficiency (POI) patients, and that PGRMC1 mRNA and protein levels were also significantly upregulated in HA-treated cells [89].

HMWHA plays a potential role in preventing bacterial ascension through the cervical canal, thereby reducing the risk of preterm birth (PTB). Scientific evidence indicates that the depletion of HMWHA is associated with the structural disorganization of the cervical epithelium, which may facilitate bacterial ascension through the cervical canal leading to inflammation and/or infection and ultimately increasing the likelihood of PTB [101,102,103], Studies focusing on Group B *Streptococci* (GBS) highlighted that the loss of HMWHA results in a higher increase in the incidence of ascending infections [104]. GBS produces hyaluronidases that degrade HMWHA into low-molecular-weight fragments, which bind to Tool-Like Receptors 2/4 (TLR 2/4), triggering a pro-inflammatory response [105]. Unlike LMWHA, intact HMWHA polymers shield TLRs, thereby preventing the activation of inflammatory cascades [106]. Lee, B.M. et al. reported that a high concentration of HMWHA significantly decreased nitric oxide (NO) production by lipopolysaccharide (LPS)-stimulated macrophages, decreased the expression of genes associated with classically activated (M1) macrophages (such as TNF-α, IL-6, CCL2, and IL-1β), and upregulated the expression of genes linked with anti-inflammatory responses (M2 phenotype), such as TGF-β1, IL10, IL-11, and Arg1 [106]. Recently, an in vivo study investigated the efficacy of oral HMWHA supplementation in attenuating experimentally induced PTB in female Wistar rats. The findings demonstrated that HMWHA administration effectively downregulated pro-inflammatory cytokines TNF-α and IL-1β, suggesting its protective role against inflammation-mediated PTB [107].

### 3.4. HMWHA Helps in Counteracting Adverse Events in Pregnancy

In general, oral supplements are convenient and affordable, easy to integrate into daily routines, and cost-effective for long-term use. They are available in various forms tailored to specific health conditions. When orally ingested, HA, depending on its molecular weight, is first exposed to an acidic environment in the stomach, where it degrades due to the pH. HMWHA does not significantly degrade when passing through the stomach system, in contrast to LMWHA [108,109]. Regarding this, Kimura et al. showed that HMWHA was not broken down by artificial gastric or intestinal juices, but that HA oligomers with fewer than six units could be further broken down and absorbed in the cecum [109]. Since it is hard to pinpoint exactly where HA is distributed throughout the body, the absorption of HA is still debatable. Because of this, HA absorption using radiolabeled molecules has been assessed in several investigations, thus clarifying that in the intestinal tract, HA uptake can follow different routes [110]. While several studies reported the ability of vLMWHA to permeate through the intestinal barrier, passively passing through paracellular pathways, this is not applicable to HA with a molecular weight >10 kDa, which is absorbed in different ways [111]. Even though several cells (including dendritic cells, macrophages, M cells, and enterocytes) absorb HMWHA after consumption, only M cells can transport intact HMWHA to the gut-associated lymphatic tissue because of their unique lysosome paucity and acid phosphatase deficiency. Evidence from experiments has previously demonstrated that other GAGs do not break down when taken orally and are eliminated in an unaltered state in urine. Furthermore, contrary to what would be predicted if HA were transformed or metabolized into tiny HA oligosaccharides, oral administration trials using HMWHA did not demonstrate pro-inflammatory effects [111].

Clinical evidence about the important role of oral HMWHA in supporting pregnancy evolution has been emerging in recent years [112,113]. The first study was a retrospective observational study in which data from a total of about 250 pregnant women, aged between 25 and 41 years old, at the 7th gestational week, were collected. A total of *n* = 200 pregnant women were given oral supplement tablets containing 200 mg HMWHA, in combination with other natural molecules (ALA, magnesium, vitamin B6, and vitamin D). The percentage of adverse events, such as miscarriage, preterm birth (*p* = 0.0092), pelvic pain (*p* < 0.0001), uterine contractions (*p* = 0.0394), and hospitalization (*p* < 0.0001) was significantly lower in the treatment group compared with the control group [112].

Oral HMWHA supplementation has also been demonstrated to be effective in the case of threatened miscarriage, a widespread complication in the first 20 weeks of gestation, affecting 20–25% of pregnancies [114]. Pregnant women with threatened miscarriage can simultaneously experience abdominal cramps, pelvic pain, pelvic pressure, and/or back pain [114]. Currently, the known causes of threatened miscarriage include changes in the levels of cytokines and placental membranes, maternal immune dysfunction, and endocrine abnormalities [115]. In cases of threatened miscarriage, pregnant women can often experience a subchorionic hematoma (SCH), a typical anomaly during gestation, defined as the collection of blood between the chorionic membrane and the uterine wall [116]. Although SCH is common during gestation, with an incidence of up to 39.5%, it may correlate with a 46% risk of several adverse pregnancy outcomes, including PTB, PPROM, and early and late pregnancy loss [117]. Therefore, recovering from SCH is an essential goal to prevent miscarriage.

A recent clinical trial [113] investigated if oral HMWHA could promote SCH resorption and resolve related symptoms (vaginal bleeding abdominal pain, and uterine contractions) in pregnant women with threatened miscarriage.

The study enrolled 56 pregnant women with a gestational age between the 6th and 13th weeks. Participants were assigned to either a control group (*n* = 25), receiving vaginal progesterone (200 mg twice daily), or a treatment group (*n* = 31), receiving the same progesterone regimen plus HMWHA (200 mg) combined with other natural compounds for two weeks.

The primary outcome of the study was the reduction in/disappearance of subchorionic hematoma. The secondary outcome was the reduction in maternal subjective symptoms such as pelvic pain, vaginal bleeding, and uterine contractions. Results demonstrated that HMWHA, in combination with natural molecules, significantly accelerated the resorption of subchorionic hematoma and symptom resolution compared with the control group [113].

**Table 2:** Experimental and clinical evidence about the use of HMWHA to support physiological pregnancy.

Study	Model and Design	Interventions	Findings	Ref.
Nakagawa, K. et al., 2012	Fresh embryo transfer (fresh ET)	0.5 mg/mL of hyaluronan or control medium	High concentration of HA supports the embryo during initial implantation into the endometrium	[75]
Nakamura, K. et al., 2004	Mice (in vivo study)	0.35% HMWHA in 0.5 ml solution versus vehicle	HMWHA inhibits pro-inflammatory factors (TNF-α or IFN-γ)	[77]
Wang, S. et al., 2019	Trophoblast cells (Tros)	HMWHA (50 or 100 μg/mL)	HMWHA induces M2 polarization of macrophages at the maternal–fetal interface	[79]
Zhao, G. et al., 2014	POI patients	Plasma endogenous levels of HMWHA	HMWHA correlates with PGRMC1 expression	[89]
Zhao, G. et al., 2014	Granulosa cells (in vitro study)	HMWHA (100 µg/mL, 200 µg/mL, and 500 µg/mL)	HMWHA increases PGRMC1 expression in a time- and concentration-dependent manner	[89]
Cilaker Micili, S. et al., 2023	Rats (in vivo study)	Low dose (2.5 mg) and high dose (5 mg)	HMWHA prevents PTB and decreases inflammatory cytokines (IL-1β and TNF-α)	[107]
Parente, E. et al., 2023	Pregnant women (clinical study)	HMWHA (200 mg) in association with natural molecules versus control group	HMWHA prevents PTB and adverse events (pelvic pain, spontaneous contractions, miscarriages, and hospitalization)	[112]
Porcaro, G. et al., 2024	Pregnant women (clinical study)	HMWHA (200 mg) in association with natural molecules in association with vaginal P4 versus control group	HMWHA induces SCH resorption faster and improves related symptoms (vaginal bleeding, abdominal pain, and uterine contractions)	[113]

POI = primary ovarian insufficiency; PTB = preterm birth; SCH = subchorionic hematoma; and P4 = progesterone.

## 4. Conclusions

**Figure 2:**
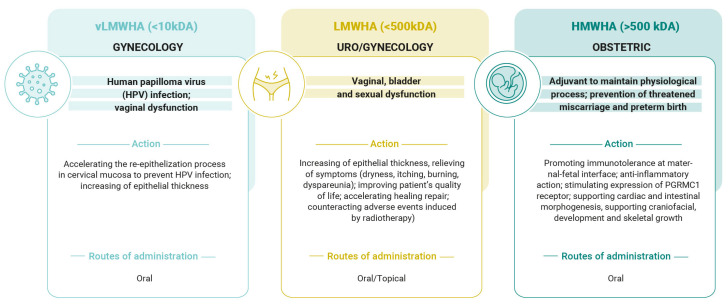
Principal actions of hyaluronic acid based on its molecular weight in gynecology and obstetrics fields.

All living things contain hyaluronic acid, an intriguing natural chemical with a variety of characteristics based on its molecular weight. These factors make HA suitable for a variety of medical uses. Most significantly, it can be applied to obstetrics and gynecology to enhance women’s health at various stages of life, as summarized in Figure 2.

LMWHA supports ECM remodeling and tissue regeneration by inducing the synthesis of growth factors, chemokines, and pro-inflammatory cytokines. Because of this characteristic, LMWHA and vLMWH are very helpful molecules in counteracting cervical and uro/gynecological diseases, such as in cases of HPV infection, menopause, and pelvic radiotherapy. Conversely, HMWHA possesses significant immunomodulatory and anti-inflammatory qualities, which are pivotal for pregnancy evolution. HMWHA is involved in organogenesis, embryo implantation, and trophoblast migration. Moreover, it enhances cervical competence by preventing bacterial ascension through the cervical canal, reducing the risk of preterm birth; supports endogenous P4 activity; and preserves immunotolerance at the maternal/fetal interface. Hyaluronic acid is a very peculiar molecule, since it can be used alone or in association with other natural molecules. In our paper, we have mentioned, for example, that in the case of human papilloma virus (HPV) infection, very low-molecular-weight hyaluronic acid has been associated with epigallocatechin gallate (EGCG) + folic acid (FA) + vitamin B12 (B12), thus inducing a significant increase in apoptosis and p53 gene expression in HPV-infected cells and concomitantly decreasing viral E6/E7 gene expression. Clinically, this association causes a significant decrease in cervical and anal lesions and improves HPV viral clearance. In light of these properties, it could certainly be desirable to extend its use in countries where, due to low levels of economic opportunity, there is no possibility of access to vaccinations in the context of HPV infections [118].

In the obstetric field, HMWHA, alone or in association with vitamin D, magnesium, or vitamin B6, may prevent pregnancy complications, such as miscarriage, preterm birth, pelvic pain, and uterine contractions.

Given the advantages of high-molecular-weight hyaluronic acid in the treatment of pregnancy-related issues, like preterm birth, its application would be ideal in low-socio-demographic-index (SDI) regions, where the prevalence of this complication is still very high despite global trends [119].

In general, hyaluronic acid is safe and well tolerated; no contraindications or disadvantages currently exist for its use. Particularly, when considering the articles cited in the paper, no adverse events or side effects have been reported in connection with its use.

Considering this, we expect that more clinical trials will examine the role of the different molecular weights in the many domains of use, given the molecules’ immense potential and safety.

## Data Availability

No new data were created in this review article. Data sharing is not applicable to this review.
